# Supplementary material for: Bayesian refinement of protein structures and ensembles against SAXS data using molecular dynamics
Source: PLoS Comput Biol. 2017 Oct 18;13(10):e1005800. doi: 10.1371/journal.pcbi.1005800 (PMC5662244; doi:10.1371/journal.pcbi.1005800)
Supplement: S1 Archive — (BZ2) [file pcbi.1005800.s011.tar.bz2 › gromacs_4.62_bayesian_saxs_refi/share/html/online/mk_angndx.html]

mk\_angndx


| mk\_angndx Main Table of Contents |

**VERSION 4.6  
Sat 19 Jan 2013**

---

### Description

mk\_angndx makes an index file for calculation of
angle distributions etc. It uses a run input file (.tpx) for the
definitions of the angles, dihedrals etc.

### Files

| option | filename | type | description |
| --- | --- | --- | --- |
| **-s** | topol.tpr | Input | Run input file: tpr tpb tpa |
| **-n** | angle.ndx | Output | Index file |

### Other options

| option | type | default | description |
| --- | --- | --- | --- |
| **-[no]h** | bool | no | Print help info and quit || **-[no]version** | bool | no | Print version info and quit || **-nice** | int | 0 | Set the nicelevel || **-type** | enum | angle | Type of angle: angle, dihedral, improper or ryckaert-bellemans || **-[no]hyd** | bool | yes | Include angles with atoms with mass < 1.5 || **-hq** | real | -1 | Ignore angles with atoms with mass < 1.5 and magnitude of their charge less than this value |

---

http://www.gromacs.org  
gromacs@gromacs.org
